# Supplementary material for: Detection of Frustration-related Operant Behavior in Rats via Machine Learning Methods
Source: bioRxiv. 2026 Sep 1:2026.08.26.747319. Preprint. [Version 1] doi: 10.64898/2026.08.26.747319 (PMC13684545; doi:10.64898/2026.08.26.747319)
Supplement: Supplementary file 1 [file media-1.pdf]

Supplemental Tables

Table S1: Description of Bar press features.

Feature	Description
Max force	Maximum force (g) within a bar press.
Force variation rate	Maximum absolute differences between two consecutive force signals in the bar pr
Average first 5 values	Mean of the first five force samples after press onset.
Average last 5 values	Mean of the last five force samples before press offset.
Peak sharpness	peak's prominence divided by its width at half-prominence.
Skewness	Skewness of the force samples within a press.
Kurtosis	Kurtosis of the force samples within a press.
Press duration	Total time (s) the force trace stays above the press threshold.
Peak Number	Count of detected peaks within a press.
Peak width	Duration (s) associated with detected peaks within a press.

Table S2: hyperparameters in `find_peaks`

Hyperparameter	Definition	Values searched	Optimal value
prominence	How much a peak stands out from neighboring samples.	{0.2, 0.5, 1, 2, 5, 10}	10
height	Minimum required peak amplitude.	{1, 2, 5, 10, 20, 50}	20
distance	Minimum number of samples between adjacent peaks.	{1, 3, 5, 10, 20, 50}	10
width	Minimum peak width.	{1, 2, 3, 5, 10, 20}	5

Table S3: Hyperparameter search space and selected values for the gradient boosting model.

Hyperparameter	Values Searched	Selected Value
max_depth	$\{1, 3, 5, 7, 9\}$	3
subsample	$\{0.6, 0.75, 0.9, 1.0\}$	1.0
colsample_bytree	$\{0.6, 0.75, 0.9, 1.0\}$	0.6
reg_lambda	$\{0, 0.1, 0.5, 1, 3, 10\}$	0.1
